# State Laws Prohibiting Sales to Minors and Indoor Use of Electronic Nicotine Delivery Systems — United States, November 2014

**Published:** 2014-12-12

**Authors:** Kristy Marynak, Carissa Baker Holmes, Brian A. King, Gabbi Promoff, Rebecca Bunnell, Timothy McAfee

**Affiliations:** 1 Office on Smoking and Health, National Center for Chronic Disease Prevention and Health Promotion, CDC

Electronic nicotine delivery systems (ENDS), including electronic cigarettes (e-cigarettes) and other devices such as electronic hookahs, electronic cigars, and vape pens, are battery-powered devices capable of delivering aerosolized nicotine and additives to the user. Experimentation with and current use of e-cigarettes has risen sharply among youths and adults in the United States (1,2). Youth access to and use of ENDS is of particular concern given the potential adverse effects of nicotine on adolescent brain development (3). Additionally, ENDS use in public indoor areas might passively expose bystanders (e.g., children, pregnant women, and other nontobacco users) to nicotine and other potentially harmful constituents (4,5). ENDS use could have the potential to renormalize tobacco use and complicate enforcement of smoke-free policies (1). State governments can regulate the sales of ENDS and their use in indoor areas where nonusers might be involuntarily exposed to secondhand aerosol (4,5). To learn the current status of state laws regulating the sales and use of ENDS, CDC assessed state laws that prohibit ENDS sales to minors and laws that include ENDS use in conventional smoking prohibitions in indoor areas of private worksites, restaurants, and bars. Findings indicate that as of November 30, 2014, 40 states prohibited ENDS sales to minors, but only three states prohibited ENDS use in private worksites, restaurants, and bars. Of the 40 states that prohibited ENDS sales to minors, 21 did not prohibit ENDS use or conventional smoking in private worksites, restaurants, and bars. Three states had no statewide laws prohibiting ENDS sales to minors and no statewide laws prohibiting ENDS use or conventional smoking in private worksites, restaurants, and bars. According to the Surgeon General, ENDS have the potential for public health harm or public health benefit (1). The possibility of public health benefit from ENDS could arise only if 1) current smokers use these devices to switch completely from combustible tobacco products and 2) the availability and use of combustible tobacco products are rapidly reduced (1). Therefore, when addressing potential public health harms associated with ENDS, it is important to simultaneously uphold and accelerate strategies found by the Surgeon General to prevent and reduce combustible tobacco use, including tobacco price increases, comprehensive smoke-free laws, high-impact media campaigns, barrier-free cessation treatment and services, and comprehensive statewide tobacco control programs (1).

Data on state laws enacted as of November 30, 2014, were obtained from CDC’s State Tobacco Activities Tracking and Evaluation (STATE) System for the 50 states and the District of Columbia.* STATE contains tobacco-related state laws collected quarterly from the LexisNexis online legal research database.† This study examined laws that explicitly prohibit: 1) ENDS sales to minors; and 2) ENDS use in indoor areas of private-sector worksites, restaurants, and bars. Laws that made general reference to tobacco products or tobacco consumption, without explicit reference to ENDS, were excluded. State laws covering private-sector worksites, restaurants and bars were assessed to determine whether these laws align with CDC’s definition of a comprehensive smoke-free law (i.e., prohibiting smoking in all indoor areas of private worksites, restaurants, and bars) (6). U.S. Census Bureau estimates as of July 2013 were used to estimate population coverage.§

A total of 40 state laws prohibit ENDS sales to minors (Table); sales are prohibited to persons aged <18 years in 36 states and <19 years in Alabama, Alaska, New Jersey, and Utah (Figure 1). Twelve states enacted such laws effective during 2010–2012, compared with 12 states in 2013, and 16 states by November 30, 2014 (Table). Approximately 16 million children aged <18 years can legally purchase ENDS in the remaining 11 states, including the District of Columbia.

Whereas 27 states, including the District of Columbia, have comprehensive smoke-free laws that prohibit smoking in restaurants, worksites, and bars, only three limit indoor ENDS use: New Jersey, North Dakota, and Utah (Figure 2). Thus, an estimated 303 million U.S. residents, including 70 million children, live in states in which nonusers of these products can be passively exposed to either secondhand smoke from cigarettes and other combustible tobacco products or ENDS aerosol. No states have enacted comprehensive smoke-free laws or laws prohibiting ENDS use in private worksites, restaurants, and bars since 2012 (Table).

Two states (New Jersey and Utah) prohibit ENDS sales to minors and indoor smoking and indoor ENDS use in private worksites, restaurants, and bars (Table). Three states (Nevada, Pennsylvania, and Texas) have neither type of law (Table). Among the 40 states with laws prohibiting ENDS sales to minors, 21 lack laws that prohibit conventional smoking and ENDS use indoors in private worksites, restaurants, and bars (Table).

## Discussion

An increasing number of states have enacted laws prohibiting ENDS sales to minors, but 11 states, including the District of Columbia, have not. Far fewer states have passed laws prohibiting ENDS use indoors, and no states have enacted such laws since 2012. The comparative lack of laws prohibiting ENDS use indoors could be attributable to limited knowledge about the potential health effects of public ENDS use and to the complexities of modifying existing state smoke-free laws (1).

Prohibitions on ENDS use in public places might be beneficial in multiple ways. First, prohibitions could preserve clean indoor air because ENDS aerosol can contain harmful and potentially harmful constituents, including nicotine and other toxins (4,5,7), and some ENDS can be modified to deliver marijuana and other psychoactive substances (8). Second, based on the experience that smoke-free policies result in diminished social acceptability of smoking (9), restrictions on ENDS use in public might help support tobacco-free norms. Third, such restrictions could support smoke-free law enforcement because some ENDS use can be difficult to distinguish from conventional smoking, thus complicating smoke-free policy enforcement. Accordingly, it is important that efforts to integrate ENDS into smoke-free laws uphold or strengthen, not weaken, existing protections against secondhand smoke exposure (1,3).

The relatively rapid adoption of laws prohibiting ENDS sales to minors compared with the slow adoption of laws prohibiting ENDS use in public indoor spaces might be attributable, in part, to the tobacco industry, which has actively advocated for state legislation to prevent minors from purchasing ENDS.¶ This is of concern because industry-supported youth-access bills have contained provisions that undermine youth prevention efforts, including preemption of stricter local policies and weak enforcement requirements (9). Additionally, laws prohibiting sales to minors are likely to have limited effectiveness as a youth tobacco prevention strategy if not coupled with proven interventions such as comprehensive smoke-free laws (1,9). Thus, among the 21 states that have laws prohibiting sales of ENDS to minors but do not have comprehensive smoke-free laws, protections against the use of conventional tobacco and ENDS in indoor public places would benefit public health. Laws prohibiting sales of ENDS to minors that allow for local action and feature strong enforcement provisions are more likely to help prevent youth access (9).

The recent rapid increase in ENDS use by youth and adults might be partially attributable to increased advertising of these products, particularly on television (10). Some marketing suggests that ENDS can be used in places where smoking is not allowed (1) or refers customers to advocacy groups that oppose indoor ENDS use prohibitions (3).**†† These groups contend that ENDS emit fewer toxins than combustible tobacco, and that public use could encourage smokers to switch to ENDS. However, ENDS aerosol is not as safe as clean air. Nicotine is a psychoactive chemical with known harms and irritant effects (1). Research has documented the presence of secondhand nicotine exposure using environmental monitoring and the measurement of biomarkers among exposed nonusers (5,7). Moreover, public ENDS use might prolong smoking by facilitating situational substitution of ENDS when smoking is not allowed, rather than complete substitution (1,3). Using a public health standard, policies should consider potential adverse impacts on the entire population, particularly children and nonusers (1).

The Food and Drug Administration (FDA) has issued a proposal to regulate additional products meeting the legal definition of a tobacco product, including ENDS, through authority granted by the Family Smoking Prevention and Tobacco Control Act.§§ If finalized as written, the rule would establish, among other provisions: restrictions to prevent sales to minors, to prohibit free samples, and to prohibit vending machine sales, unless in a facility that never admits minors. The proposed rule must undergo several steps before becoming final, and during this period there could be further increases in youth ENDS use. Furthermore, FDA regulation will not address certain key policy interventions related to ENDS, such as use in public places. The Family Smoking Prevention and Tobacco Control Act allows states and localities to adopt or continue to enforce additional or more stringent requirements than those stipulated. Additional national and state policies addressing retailer licensing, Internet sales, taxation, and marketing could further prevent youth use of ENDS and other tobacco products (1,3).

What is already known on this topic?

Electronic nicotine delivery systems (ENDS), including electronic cigarettes (e-cigarettes), are battery-powered devices capable of delivering aerosolized nicotine and other additives to the user. State governments play an integral role in regulating the sales of ENDS and ensuring that citizens are protected from involuntary exposure to secondhand smoke, nicotine, and other potentially harmful constituents.

What is added by this report?

As of November 30, 2014, 40 states have enacted laws prohibiting ENDS sales to minors, but only three of the 27 states with comprehensive smoke-free air laws have incorporated ENDS. Approximately 16 million children can legally purchase ENDS, and 303 million U.S. residents, including 70 million children, live in states in which non–tobacco users could be passively exposed to either secondhand smoke from cigarettes and other combustible tobacco products, or ENDS aerosol, in private worksites, restaurants, and bars.

What are the implications for public health practice?

When addressing potential public health harms associated with ENDS, it is critical to simultaneously uphold and accelerate strategies proven to prevent and reduce use of conventional tobacco products, including tobacco price increases, comprehensive smoke-free air laws, high-impact media campaigns, barrier-free cessation treatment and services, and comprehensive statewide tobacco control programs.

The findings in this report are subject to at least two limitations. First, STATE does not contain bills under consideration, regulations, local laws, opinions of attorneys general, or case law decisions for tobacco control topics other than preemption. Importantly, over 200 localities have included ENDS prohibitions in their comprehensive smoke-free laws.¶¶ Second, the strength of each law or the specific language contained in each law was not assessed. Statutory definitions of ENDS (e.g., as a tobacco product) vary across states. For example, some states’ statutory definitions of ENDS define the products as alternative nicotine or vapor products that are exempt from regulations or taxes that apply to tobacco products. Including ENDS in the state or local statutory definition of tobacco products could facilitate the extension of additional tobacco control policies to ENDS, such as retailer licensing requirements, taxation, and marketing provisions.

Although ENDS might have the potential to benefit established adult smokers if used as a complete substitute for all combusted tobacco products, ENDS should not be used by youths and adult nontobacco users because of the adverse effects of nicotine and other risk exposures, as well as the risk for progression to other forms of tobacco use (1). The findings in this report suggest that states have additional opportunities to prevent access to ENDS, avoid renormalization of tobacco use, and preserve clean indoor air standards.

Proven tobacco prevention strategies, including comprehensive smoke-free laws and robust prohibitions against sales to minors, could be effective in preventing youth ENDS use and denormalizing tobacco use. Simultaneously upholding and accelerating strategies proven to prevent conventional tobacco use, support tobacco cessation, and prevent secondhand smoke exposure would benefit public health (1,3).

## Figures and Tables

**FIGURE 1:**
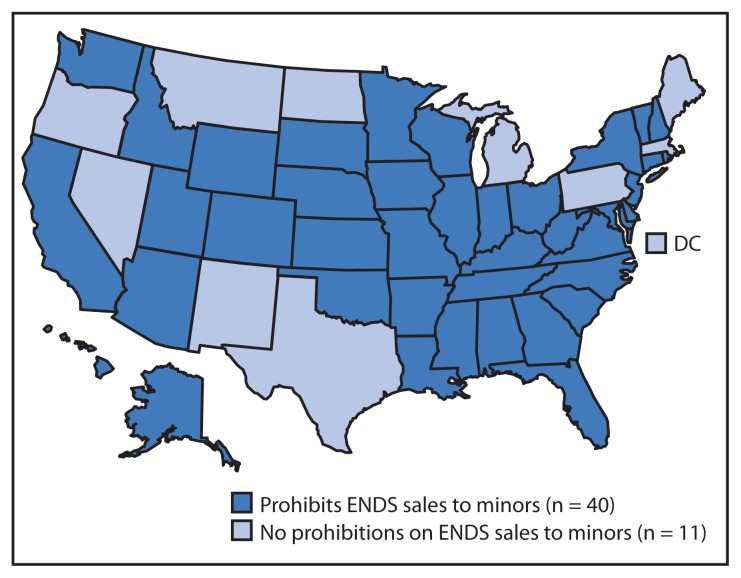
States with and without laws prohibiting sales of electronic nicotine delivery systems (ENDS) to minors^*^ — United States, November 30, 2014 ^*^ Minors are defined by statute as persons aged <18 years, except in four states where they are defined as persons aged <19 years (Alabama, Alaska, New Jersey, and Utah).

**FIGURE 2:**
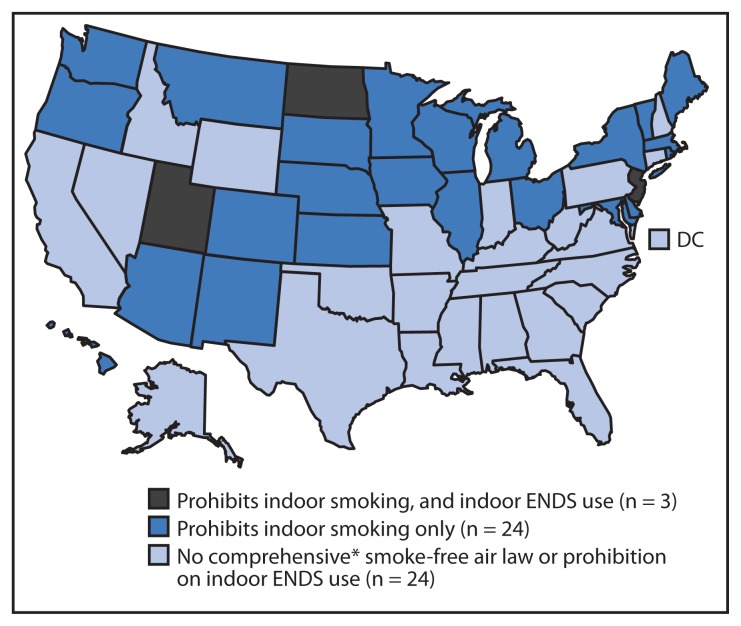
States with and without laws prohibiting smoking and use of electronic nicotine delivery systems (ENDS) in indoor areas of private worksites, restaurants, and bars — United States, November 30, 2014 ^*^ CDC defines a state smoke-free air law as comprehensive if it prohibits smoking in indoor areas of private worksites, restaurants, and bars.

**TABLE:** State laws prohibiting sales of electronic nicotine delivery systems (ENDS) to minors and laws prohibiting conventional smoking and the use of ENDS in indoor areas of private worksites, restaurants, and bars — United States, November 30, 2014

State	Effective date of law restricting ENDS sales to minors (minimum age allowed [yrs])	State smoke-free law		Summary of laws enacted as of November 30, 2014

		Prohibits conventional smoking in worksites, restaurants, and bars (effective date)	Includes restriction on ENDS use (effective date)	
Alabama	8/1/2013 (19)			YA
Alaska	8/22/2012 (19)			YA
Arizona	9/13/2013 (18)	5/1/2007	No	YA/SF
Arkansas	8/16/2013 (18)			YA
California	9/27/2010 (18)			YA
Colorado	3/25/2011 (18)	7/1/2006	No	YA/SF
Connecticut	10/1/2014 (18)			YA
Delaware	6/12/2014 (18)	12/1/2002	No	YA/SF
District of Columbia		1/1/2007	No	SF
Florida	7/1/2014 (18)			YA
Georgia	7/1/2014 (18)			YA
Hawaii	6/27/2013 (18)	11/16/2006	No	YA/SF
Idaho	7/1/2012 (18)			YA
Illinois	1/1/2014 (18)	1/1/2008	No	YA/SF
Indiana	7/1/2013 (18)			YA
Iowa	7/1/2014 (18)	7/1/2008	No	YA/SF
Kansas	7/1/2012 (18)	7/1/2010	No	YA/SF
Kentucky	4/10/2014 (18)			YA
Louisiana	5/28/2014 (18)			YA
Maine		9/11/2009	No	SF
Maryland	10/1/2012 (18)	2/1/2008	No	YA/SF
Massachusetts		7/5/2004	No	SF
Michigan		5/1/2010	No	SF
Minnesota	8/1/2010 (18)	10/1/2007	No	YA/SF
Mississippi	7/1/2013 (18)			YA
Missouri	9/10/2014 (18)			YA
Montana		10/1/2009	No	SF
Nebraska	4/9/2014 (18)	6/1/2009	No	YA/SF
Nevada				
New Hampshire	7/31/2010 (18)			YA
New Jersey	3/12/2010 (19)	4/15/2006	Yes (7/11/2010)	YA/SF/EF
New Mexico		6/15/2007	No	SF
New York	1/1/2013 (18)	7/24/2003	No	YA/SF
North Carolina	8/1/2013 (18)			YA
North Dakota		12/6/2012	Yes (12/6/2012)	SF/EF
Ohio	8/2/2014 (18)	12/7/2006	No	YA/SF
Oklahoma	11/1/2014 (18)			YA
Oregon		1/1/2009	No	SF
Pennsylvania				
Rhode Island	6/30/2014 (18)	3/1/2005	No	YA/SF
South Carolina	6/7/2013 (18)			YA
South Dakota	7/1/2014 (18)	11/10/2010	No	YA/SF
Tennessee	7/1/2011 (18)			YA
Texas				
Utah	5/11/2010 (19)	1/1/2009	Yes (5/8/2012)	YA/SF/EF
Vermont	7/1/2013 (18)	7/1/2009	No	YA/SF
Virginia	7/1/2014 (18)			YA
Washington	7/28/2013 (18)	12/8/2005	No	YA/SF
West Virginia	6/27/2014 (18)			YA
Wisconsin	4/20/2012 (18)	7/5/2010	No	YA/SF
Wyoming	3/13/2013 (18)			YA
**Total**	**40**	**27**	**3**	**N/A**

**Abbreviations:** YA = youth access (state law prohibits sales of ENDS to minors); SF = smoke-free (state has a comprehensive smoke-free law that prohibits smoking in indoor areas of private worksites, restaurants, and bars; EF = ENDS-free (state law prohibits the use of ENDS in indoor areas of private worksites, restaurants, and bars).

## Footnotes

* Additional information available at http://www.cdc.gov/tobacco/statesystem. (ENDS laws will be added to the STATE System database in 2015.)

† Additional information available at http://www.lexisnexis.com.

§ Additional information available at http://factfinder2.census.gov/faces/tableservices/jsf/pages/productview.xhtml?pid=PEP_2013_PEPAGESEX&prodType=table.

¶ Additional information available at http://www.commerce.senate.gov/public/?a=Files.Serve&File_id=7e32673d-795b-44ce-979b-cc9d6c4f1d48.

** Additional information available at http://www.howtonjoyvaping.com/home-1.

†† Additional information available at http://sfata.org/calls-to-action.

§§ Additional information available at http://www.gpo.gov/fdsys/pkg/PLAW-111publ31/content-detail.html.

¶¶ Additional information available at http://www.no-smoke.org/pdf/ecigslaws.pdf.
